# Activity of Aztreonam/Avibactam and Recently Approved β-Lactamase Inhibitor Combinations against Enterobacterales and *Pseudomonas aeruginosa* from Intensive Care Unit and Non-Intensive Care Unit Patients

**DOI:** 10.3390/antibiotics13060564

**Published:** 2024-06-17

**Authors:** Helio S. Sader, Rodrigo E. Mendes, John H. Kimbrough, Cory M. Hubler, Mariana Castanheira

**Affiliations:** Element Iowa City (JMI Laboratories), 345 Beaver Kreek Ctr, Ste A, North Liberty, IA 52240, USA; rodrigo.mendes@element.com (R.E.M.); hank.kimbrough@element.com (J.H.K.); mariana.castanheira@element.com (M.C.)

**Keywords:** ceftazidime/avibactam, ceftolozane/tazobactam, meropenem/vaborbactam, *Pseudomonas aeruginosa*, carbapenem-resistant Enterobacterales

## Abstract

We evaluated the activities of aztreonam/avibactam and recently approved β-lactamase inhibitor combinations (BLICs) to compare the antimicrobial susceptibility patterns of Enterobacterales and *Pseudomonas aeruginosa* isolated from intensive care unit (ICU) and non-ICU patients. Clinical isolates (1/patient) were consecutively collected from 72 United States medical centres in 2020–2022 and susceptibility tested by broth microdilution. The results for 5421 isolates from ICU patients were analysed and compared to those for 20,649 isolates from non-ICU patients. Isolates from ventilator-associated pneumonia patients were analysed separately. Aztreonam/avibactam inhibited 100.0%/>99.9% Enterobacterales and 100.0%/98.3% of carbapenem-resistant Enterobacterales (CRE) from ICU/non-ICU patients at ≤8 mg/L, respectively. The CRE susceptibility rates were 88.5%/82.9% for ceftazidime/avibactam, 82.1%/81.2% for meropenem/vaborbactam, and 78.2%/72.6% for imipenem/relebactam among ICU/non-ICU isolates. Among the *P. aeruginosa* isolates from ICU/non-ICU patients, the susceptibility rates were 96.3%/97.6% for ceftazidime/avibactam, 97.2/98.4% for ceftolozane/tazobactam, 97.1%/98.0% for imipenem/relebactam, 77.8%/84.6% for piperacillin/tazobactam, and 76.9%/85.8% for meropenem; aztreonam/avibactam inhibited 78.0%/81.9% of *P. aeruginosa* at ≤8 mg/L. In summary, lower susceptibility rates were observed among ICU than non-ICU isolates. Aztreonam/avibactam exhibited potent in vitro activity and broad-spectrum activity against Enterobacterales from ICU and non-ICU patients, including CRE and isolates non-susceptible to newer BLICs. Against *P. aeruginosa*, aztreonam/avibactam showed a spectrum of activity comparable to that of piperacillin/tazobactam, meropenem, and ceftazidime.

## 1. Introduction

Healthcare-associated infections represent an important challenge when treating critically ill patients. Mortality rates and the length of hospital stays are markedly higher among intensive care unit (ICU) patients with an infection compared to non-infected patients [1]. An important factor that contributes to poor patient outcomes is a delay in the introduction of effective antimicrobial therapy, which is more likely among patients infected with antimicrobial-resistant organisms than among patients infected with susceptible organisms [2,3,4].

Various factors contribute to increasing antimicrobial resistance in ICU patients, including the use of indwelling devices, the high occurrence of invasive procedures, the presence of comorbidities, prolonged hospital stays, the excessive use of antimicrobial agents, and the spread of resistant organisms [1,5]. Gram-negative organisms, mainly Enterobacterales and *Pseudomonas aeruginosa*, are responsible for most infections in United States (US) ICUs, and antimicrobial resistance is remarkably problematic among these organisms [6]. Moreover, inappropriate empiric therapy is clearly more frequent in Gram-negative than Gram-positive infections [7]. 

Multidrug-resistant (MDR) Gram-negative bacteria are the leading causes of nosocomial pneumonia, including ventilator-associated pneumonia (VAP) and bloodstream infection in ICU patients. Notably, carbapenem resistance has increased in the last decade, and it is commonly caused by carbapenemases that are able to hydrolyse most β-lactams currently available for clinical use in the US and Europe [8,9]. 

Various antimicrobial agents targeting MDR Gram-negative organisms have been licensed in recent years, including ceftazidime/avibactam, meropenem/vaborbactam, imipenem/relebactam, and cefiderocol [10]. These agents represent an important improvement in the treatment of infections caused by MDR Gram-negative organisms, especially carbapenem-resistant Enterobacterales (CRE); however, resistance to these agents appears to be increasing in some US hospitals [11].

Aztreonam/avibactam is under development to treat patients with Gram-negative infections, especially those caused by CRE and *Stenotrophomonas maltophilia*, and it has recently (April 2024) been granted marketing authorization by the European Medicines Agency (EMA) in the European Union (https://www.ema.europa.eu/en/news/new-antibiotic-fight-infections-caused-multidrug-resistant-bacteria; accessed on 6 May 2024) [12,13]. Aztreonam is stable to hydrolysis by metallo-β-lactamases (MBLs), and avibactam protects aztreonam from hydrolysis by serine β-lactamases, such as chromosomal derepressed AmpC, extended-spectrum β-lactamases, and *Klebsiella pneumoniae* carbapenemases (KPCs). In this investigation, we evaluated the in vitro activity of aztreonam/avibactam and recently approved β-lactamase inhibitor combinations (BLICs) and compared the antimicrobial susceptibility patterns of Enterobacterales and *P. aeruginosa* isolated from ICU and non-ICU patients.

## 2. Results

**Figure 1:**
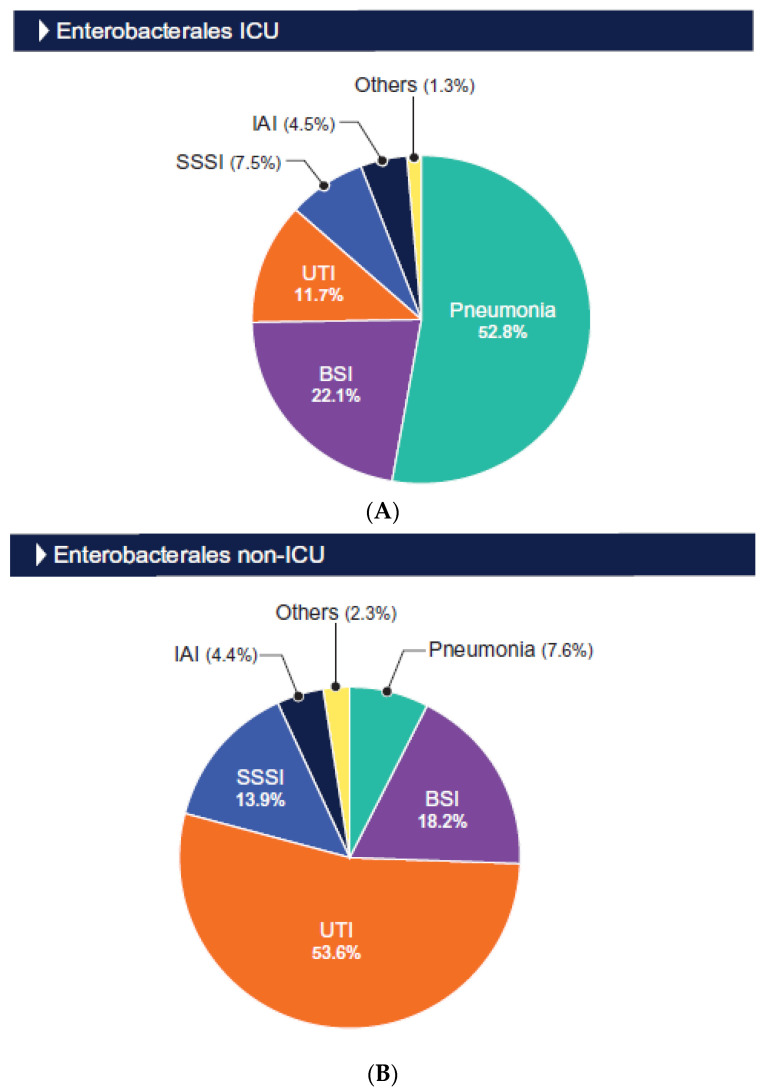
Origin of Enterobacterales isolated from ICU (**A**) and non-ICU (**B**) patients. Abbreviations: BSI, bloodstream infection; UTI, urinary tract infection; SSSI, skin and skin structure infection; IAI, intraabdominal infection.

**Figure 2:**
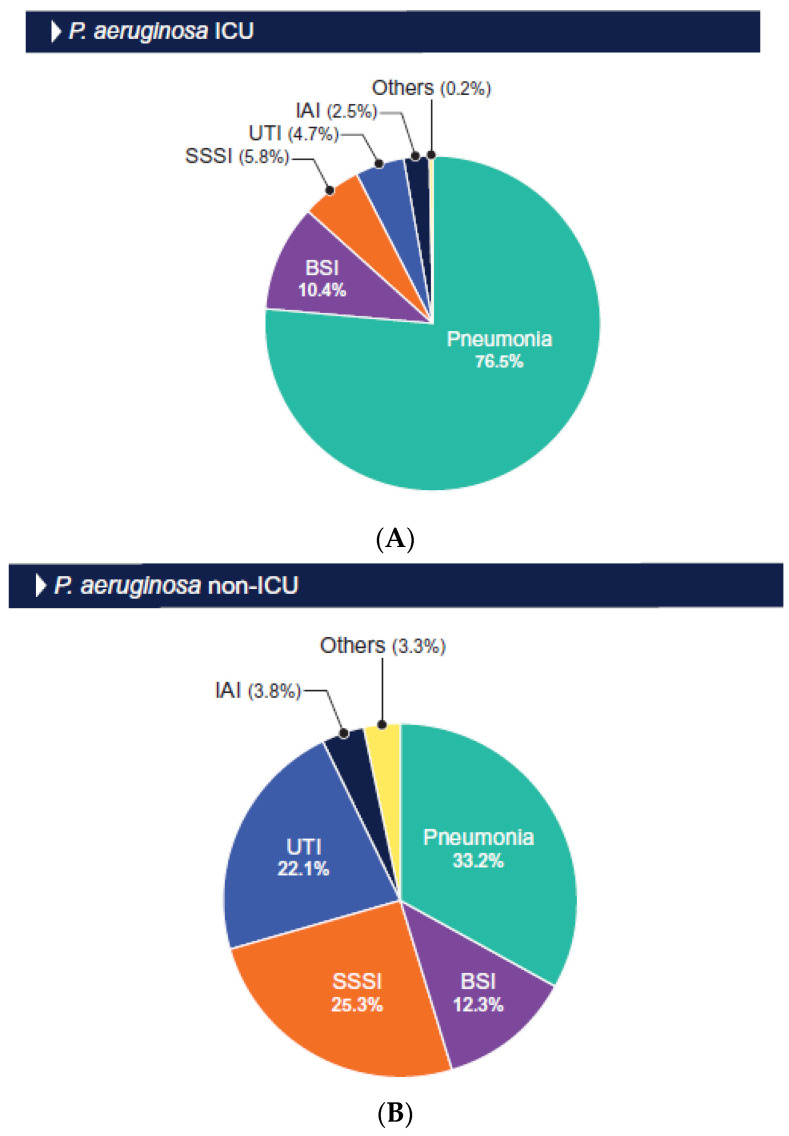
Origin of *P. aeruginosa* isolated from ICU (**A**) and non-ICU (**B**) patients. Abbreviations: BSI, bloodstream infection; UTI, urinary tract infection; SSSI, skin and skin structure infection; IAI, intraabdominal infection.

The distributions of the isolates by infection type are shown in Figure 1 and Figure 2. The Enterobacterales and *P. aeruginosa* isolates from the ICU patients were mainly from those hospitalized with pneumonia (52.8% and 76.5%, respectively) and bloodstream infection (BSI; 22.1% and 10.4%, respectively; Figure 1 and Figure 2). Among the isolates from the non-ICU patients, the Enterobacterales isolates were mainly from urinary tract infection (UTI; 53.6% and BSI (18.2%; Figure 1), and the *P. aeruginosa* isolates were predominantly from patients hospitalized with pneumonia (33.2%), skin and skin structure infection (25.3%), and urinary tract infection (22.1%; Figure 2).

*K. pneumoniae* was the most common Enterobacterales species isolated from the ICU patients (*n* = 949; 23.1%), including those with VAP (*n* = 145; 23.0%), followed by *E. coli* (*n* = 898 [21.8%] among the ICU patients and *n* = 103 [16.3%] among the patients with VAP) and *E. cloacae* species complex (*n* = 515 [12.5%] among the ICU patients and *n =* 96 [15.2%] among the patients with VAP). In contrast, *E. coli* was the most common Enterobacterales species among the non-ICU patients (*n* = 6367; 35.6%), followed by *K. pneumoniae* (*n* = 3581; 20.0%) and *E. cloacae* (*n* = 1498; 8.4%).

Aztreonam/avibactam (MIC_50_/MIC_90_, ≤0.03/0.12 mg/L; >99.9–100.0% inhibited at ≤8 mg/L), ceftazidime/avibactam (MIC_50_/MIC_90_, 0.12/0.25–0.5 mg/L; 99.5–99.9% susceptible), and meropenem/vaborbactam (MIC_50_/MIC_90_, 0.03/0.06 mg/L; 99.7–99.9% susceptible) were highly active against Enterobacterales from ICU, VAP, and non-ICU infections (Table 1). Imipenem/relebactam activity was slightly lower than the activity of those three BLICs, with susceptibility rates ranging from 91.9% for non-ICU Enterobacterales to 96.7% for Enterobacterales from VAP (Table 1). Enterobacterales susceptibility to meropenem varied from 97.9% (VAP) to 99.3% (non-ICU; Table 1). 

**Figure 3:**
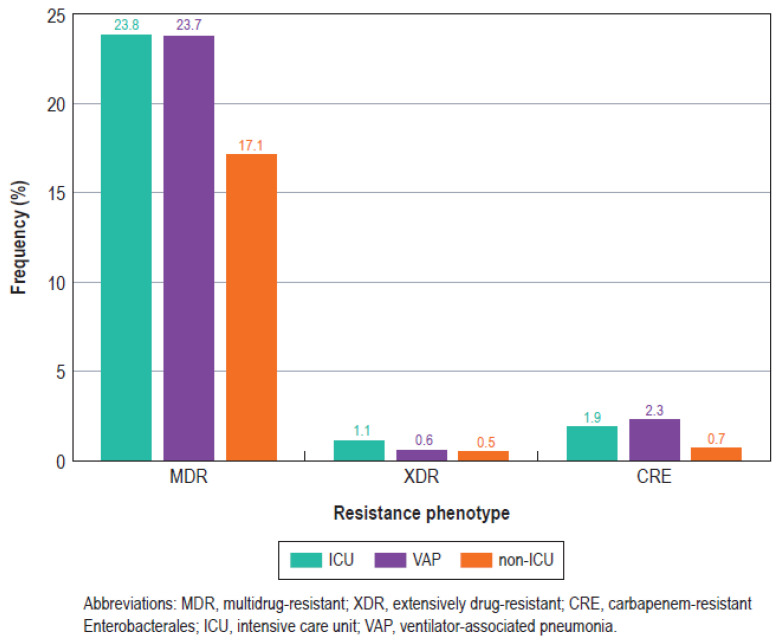
Bar graph showing the frequencies of key resistance phenotypes among Enterobacterales isolated from intensive care unit (ICU) patients, ventilator-associated pneumonia (VAP) patients, and non-ICU patients (non-ICU).

Although aztreonam/avibactam, ceftazidime/avibactam, and meropenem/vaborbactam displayed a similar spectrum of activity (% susceptible) against the Enterobacterales collected from the ICU and non-ICU patients, susceptibility for the other agents was generally lower for the ICU isolates compared to the non-ICU isolates (Table 1). For instance, the frequency of the MDR phenotype was 6.2% among the non-ICU isolates and 9.2% among the ICU isolates (7.8% for the VAP isolates; Table 1 and Figure 3). Likewise, the CRE rates were the highest among Enterobacterales from VAP (2.3%), followed by the ICU (1.9%) and non-ICU isolates (0.7%; Figure 3). The susceptibility of the three most common Enterobacterales species is shown in Table 2.

The most active compounds against the CRE isolates were aztreonam/avibactam, with 100.0% of the isolates from the ICU patients and 98.3% of the isolates from the non-ICU patients inhibited at ≤8 mg/L, followed by cefiderocol, with susceptibility rates of 92.3% and 94.9% for the ICU and non-ICU isolates, respectively (Table 1). Ceftazidime/avibactam (88.5%/82.9% susceptible for the ICU/non-ICU isolates), meropenem/vaborbactam (82.1% susceptible for the ICU and non-ICU isolates), and imipenem/relebactam (78.2%/72.6% susceptible for the ICU/non-ICU isolates) exhibited a lower spectrum of activity against the CRE isolates from both the ICU and non-ICU patients when compared to aztreonam/avibactam and cefiderocol (Table 1). 

Aztreonam/avibactam retained potent activity against MDR Enterobacterales, with 100.0% of the ICU and 99.9% of the non-ICU isolates inhibited at ≤8 mg/L (Table 1). Ceftazidime/avibactam (98.7%/99.1% susceptible for ICU/non-ICU isolates) and meropenem/vaborbactam (98.6%/99.3% susceptible for ICU/non-ICU isolates) were slightly less active against ICU Enterobacterales compared to aztreonam/avibactam. Imipenem/relebactam was active against 96.1%/96.4% of ICU/non-ICU MDR Enterobacterales, whereas ceftolozane/tazobactam exhibited limited activity against both ICU (58.6% susceptible) and non-ICU (73.7% susceptible) MDR Enterobacterales (Table 1).

Notably, aztreonam/avibactam retained activity against isolates resistant or non-susceptible to ceftazidime/avibactam (97.4% inhibited at ≤8 mg/L), meropenem/vaborbactam (100.0% inhibited at ≤8 mg/L), and imipenem/relebactam (100.0% inhibited at ≤8 mg/L; Table 3).

The most active BLICs against *P. aeruginosa* were ceftolozane/tazobactam (97.2%/98.4% susceptible for ICU/non-ICU), imipenem/relebactam (97.1%/98.0% susceptible for ICU/non-ICU), and ceftazidime/avibactam (96.3%/97.6% susceptible for ICU/non-ICU), all of which had comparable susceptibility rates (Table 1). Meropenem/vaborbactam was active against 90.0%/94.3% of the ICU/non-ICU isolates per the EUCAST criteria (there is no CLSI or US FDA breakpoint since this compound is not approved for the treatment of *P. aeruginosa* infections in the US). Aztreonam/avibactam activity against *P. aeruginosa* (78.0%/81.9% from ICU/non-ICU inhibited at ≤8 mg/L) was comparable to that of piperacillin/tazobactam (77.8%/84.6% susceptible for ICU/non-ICU), meropenem (76.9%/85.8% susceptible for ICU/non-ICU), and imipenem (77.4%/84.3% susceptible for ICU/non-ICU; Table 1).

**Figure 4:**
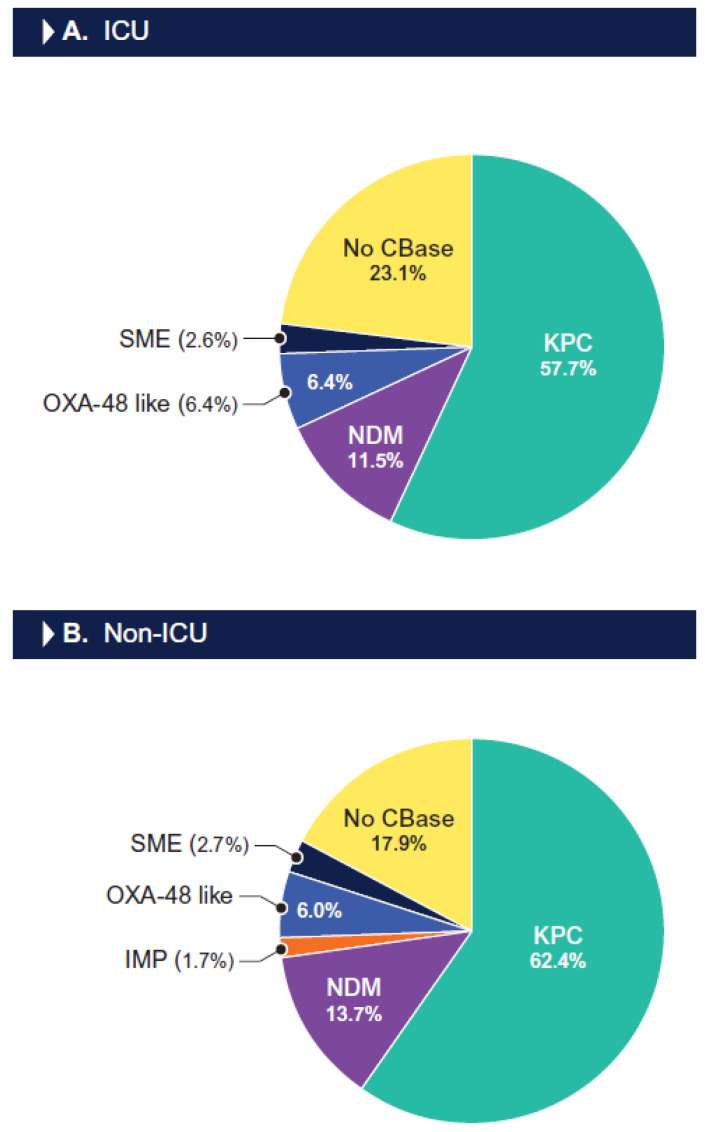
Frequency of carbapenemases among carbapenem-resistant Enterobacterales isolated from ICU (**A**) and non-ICU patients (**B**). Abbreviations: KPC, Klebsiella pneumoniae carbapenemase; NDM, New Delhi metallo-β-lactamase; IMP, imipenemase; OXA, oxacillinase; SME, Serratia marcescens enzyme; CBase, carbapenemase.

The most common carbapenemase observed among the CRE isolates from the ICU patients was the KPC type (57.7% of CREs), followed by NDM-1 (11.5%), OXA-48-like (6.4%), and SME-type (2.6%) carbapenemases. One ICU CRE isolate (1.3%) had two carbapenemases, an NDM-5 and an OXA-181. The KPC type was also the most common carbapenemase detected among the CRE isolates from the non-ICU patients (62.4%), followed by NDM-type (13.7%), OXA-48-like (6.0%), SME-type (2.7%), and IMP-type (1.7%) carbapenemases. Five non-ICU CRE isolates (4.4%) had two carbapenemases, including an IMP-4 plus a KPC-3 carbapenemase (one isolate), an NDM-type plus a KPC-3, and an NDM-type plus an OXA-48-like carbapenemase (two isolates). Notably, 11.5% of the CRE isolates from the ICU patients and 15.4% of the CRE isolates from the non-ICU patients carried an MBL, which confers resistance to ceftazidime/avibactam, meropenem/vaborbactam, and imipenem/relebactam. A carbapenemase gene was not identified in 23.1% of the CRE isolates from the ICU patients and 17.9% of the CREs from the non-ICU patients (Figure 4). Among the CRE isolates from the VAP patients (*n* = 15), a carbapenemase was observed in 10 (66.7%) isolates, including a KPC type (60.0% of CREs) and NDM-1 (6.7%).

**Figure 5:**
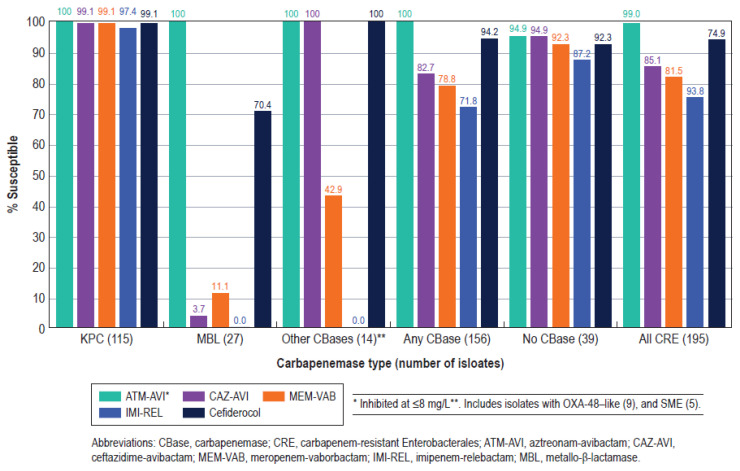
Antimicrobial activities of aztreonam/avibactam (ATM-AVI), ceftazidime/avibactam (CAZ-AVI), meropenem/vaborbactam (MEM-VAB), imipenem/relebactam (IMI-REL), and cefiderocol against carbapenemase-producing CRE stratified by main carbapenemase types among ICU and non-ICU isolates.

Aztreonam/avibactam was the most active β-lactam agent against carbapenemase-producing CRE (MIC_50_/MIC_90_, 0.25/1 mg/L; 100.0% inhibited at ≤8 mg/L), followed by cefiderocol (MIC_50_/MIC_90_, 1/4 mg/L; 94.2% susceptible), ceftazidime/avibactam (MIC_50_/MIC_90_, 1/>32 mg/L; 82.7% susceptible), meropenem/vaborbactam (MIC_50_/MIC_90_, 0.06/32 mg/L; 78.8% susceptible), and imipenem/relebactam (MIC_50_/MIC_90_, 0.25/>8 mg/L; 71.8% susceptible; Figure 5). These five compounds were very active against KPC producers, with susceptibility rates of ≥97.4%, but only aztreonam/avibactam exhibited good activity against MBL producers (Figure 5). 

## 3. Discussion

Infection is a common occurrence among patients in the ICU, and ICU-acquired infections are considered an independent risk factor for hospital mortality, even after adjustment for APACHE II or SOFA scores [16]. In a 24 h point prevalence study conducted at 1150 centres in 88 countries on 13 September 2017, 54% of patients had suspected or proven infection, and 70% of all patients were receiving prophylactic or therapeutic antimicrobial agents. Moreover, the mortality rate was 32% among patients with infection, and infection due to CRE was independently associated with an increased risk of death [17]. 

The main objective of the present study was to evaluate the antimicrobial susceptibility of Enterobacterales and *P. aeruginosa* from ICU patients in comparison to those from non-ICU patients. Although some of the recently approved β-lactam agents remain equally and highly active against ICU and non-ICU isolates, our results indicate a clear tendency of higher resistance rates among ICU than non-ICU isolates. Among Enterobacterales, the susceptibility rates were markedly lower among the ICU compared to the non-ICU isolates for ceftolozane/tazobactam (90.1% vs. 95.5%), piperacillin/tazobactam (82.4% vs. 90.5%), ceftazidime (81.7% vs. 88.1%), and ceftriaxone (77.2% vs. 84.6%). Although the susceptibility rates were similar or slightly higher among the ICU compared to the non-ICU isolates for many antimicrobial agents, the MDR, XDR, and CRE rates were clearly higher among the ICU than among the non-ICU isolates (Table 1 and Figure 3). 

Among *P. aeruginosa*, the susceptibility rates were lower among the ICU compared to the non-ICU isolates for most antimicrobial agents, with the highest differences being observed with meropenem (76.9% vs. 85.8%), imipenem (77.4% vs. 84.3%), piperacillin/tazobactam (77.8% vs. 84.6%), and ceftazidime (81.4% vs. 87.8%). Moreover, the *P. aeruginosa* isolates from the VAP patients exhibited susceptibility rates lower than the ICU isolates, especially for meropenem (70.8% vs. 76.9%), imipenem (73.7% vs. 77.4%), and piperacillin tazobactam (75.6% vs. 77.8%; Table 1).

Antimicrobial resistance rates are likely to be higher among bacteria recovered from ICU patients than non-ICU patients since various risk factors related to antimicrobial resistance are more commonly observed in ICU patients, such as the use of indwelling devices, invasive procedures, prolonged hospital stays, and a high use of antimicrobial agents; however, studies comparing the antimicrobial susceptibility rates of ICU and non-ICU isolates are scarce [1,18,19]. The results of the present study clearly show higher resistance rates among Enterobacterales and *P. aeruginosa* isolates from ICU patients and VAP patients compared to isolates from non-ICU patients.

Another important finding of this study was the reduced activity of cefiderocol and the newer β-lactamase inhibitor combinations ceftazidime/avibactam, meropenem/vaborbactam, and imipenem/relebactam against CRE and MDR Enterobacterales. Although these compounds have shown almost complete activity against US CRE and MDR Enterobacterales in previous investigations, resistance appears to be increasing recently in the US [11,20]. The main reason for the increasing resistance to these antimicrobial agents appears to be the growing prevalence of MBL-producing Enterobacterales in US medical centres, since ceftazidime/avibactam, meropenem/vaborabactam, and imipenem/relebactam are not active against MBL-producing isolates, and cefiderocol has demonstrated limited activity against NDM-producing Enterobacterales [11,21,22]. 

The limitations of this study should be contemplated when interpreting the results. The INFORM Program was not designed to assess the susceptibility profile of bacterial isolates from ICU patients in comparison to non-ICU patients; patient-unique isolates were consecutively collected unrelated to the hospital unit. After the isolates were collected and tested for susceptibility, we analysed the results of the ICU and non-ICU isolates. Variability in the criteria used to label a bacterial isolate as “clinically significant” could also be considered a study limitation, since this term was not defined in the study protocol. Instead, the criteria were determined by local algorithms, which may vary among participant medical centres. Regardless of these limitations, these results provide valuable information on the susceptibility profile of Enterobacterales and *P. aeruginosa* causing infections in the ICUs of US medical centres.

## 4. Methods

### 4.1. Organism Collection

Isolates were collected from 72 US medical centres in 2020–2022 as part of the International Network For Optimal Resistance Monitoring (INFORM) program [23]. Each participating centre was asked to collect a certain number of consecutive patient unique isolates from designated infection types during a certain period of the year, independent of the hospital unit where the patient was located. In this investigation, we assessed the susceptibility results for Enterobacterales and *P. aeruginosa* collected from ICU patients and compared them to those for isolates from patients hospitalized in other units (non-ICU). A total of 21,996 Enterobacterales isolates, comprising 4117 isolates from ICU patients and 17,879 isolates from non-ICU patients, and 4074 *P. aeruginosa* isolates, comprising 1304 isolates from ICU patients and 2770 isolates from non-ICU patients, were collected and analysed during the investigation period. The isolates were considered clinically significant by algorithms established by participant centres. Moreover, ICU isolates recovered from patients with ventilator-associated pneumonia (VAP), including 630 Enterobacterales and 308 *P. aeruginosa* isolates, were analysed separately. 

### 4.2. Susceptibility Testing

The isolates were susceptibility tested using the reference broth microdilution method, as described by CLSI [24]. Aztreonam/avibactam, ceftazidime/avibactam, ceftolozane/tazobactam, imipenem/relebactam, and piperacillin/tazobactam were tested with a β-lactamase inhibitor at a fixed concentration of 4 mg/L; meropenem/vaborbactam was tested with vaborbactam at a fixed concentration of 8 mg/L [14,24]. Cefiderocol was tested only against CRE. MIC values were interpreted according to CLSI and/or US FDA breakpoint criteria unless otherwise noted. When aztreonam/avibactam was tested against Enterobacterales, both the pharmacokinetic/pharmacodynamic (PK/PD) and EUCAST susceptible breakpoints (≤8 mg/L and ≤4 mg/L, respectively) were applied for comparison. EUCAST criteria [25] were applied for meropenem/vaborbactam against *P. aeruginosa* because this compound is not approved for the treatment of *P. aeruginosa* infections in the US, and neither CLSI nor the US FDA have published breakpoint criteria for this organism–drug combination. CLSI does not currently publish a colistin-susceptible breakpoint and categorizes isolates with an MIC ≤2 mg/L as intermediate and ≥4 mg/L as resistant; thus, the percentages of intermediate resistance are shown in Table 1. The isolates were categorized as MDR or XDR according to the criteria defined in 2012 by the joint European and US Centers for Disease Control [26]. These criteria define MDR as non-susceptible to ≥1 agent in ≥3 antimicrobial classes and XDR as susceptible to ≤2 classes. Carbapenem-resistant Enterobacterales (CRE) isolates were defined as displaying imipenem or meropenem MIC values at ≥4 mg/L. Imipenem was not applied to *Proteus mirabilis* or indole-positive Proteeae due to their intrinsically elevated MIC values. Categorical interpretations followed CLSI and/or US FDA criteria unless otherwise noted [14,15].

### 4.3. Screening for β-Lactamases

CRE isolates were tested for β-lactamase-encoding genes using Next-Generation Sequencing (NGS). Total genomic DNA was prepared using a KingFisher Cell and Tissue DNA kit (ThermoFisher Scientific, Waltham, MA, USA) or a MagMax DNA Multi-Sample Ultra 2.0 extraction kit (ThermoFisher) on a KingFisher Flex Magnetic Particle Processor (ThermoFisher). DNA libraries were constructed using either the Nextera XT library construction protocol and index kit or Illumina DNA prep (Illumina, San Diego, CA, USA), with sequencing performed on either a MiSeq Sequencer with a MiSeq Reagent Kit v3 (600 cycles) or a NextSeq 1000 Sequencer using NextSeq1000/2000 P2 Reagents (300 cycles). The generated FASTQ files were assembled using SPAdes Assembler and subjected to proprietary software version 3.15.3 (Element Iowa City [JMI Laboratories]) for the screening of β-lactamase genes [27]. 

## 5. Conclusions

Susceptibility rates were generally lower among Enterobacterales and *P. aeruginosa* isolates from ICU compared to non-ICU patients. Aztreonam/avibactam inhibited 100.0% of Enterobacterales from ICU patients and >99.9% of Enterobacterales from non-ICU patients. Against *P. aeruginosa*, the spectrum of activity for aztreonam/avibactam was comparable to that for piperacillin/tazobactam, meropenem and ceftazidime, but it was lower than that for the newer BLICs. Resistance to ceftazidime/avibactam, meropenem/vaborbactam, and imipenem/relebactam appears to be increasing among CRE but remains stable among *P. aeruginosa*, as indicated through a comparison with previous investigations. The results of the present investigation could be of great value to guide empiric antimicrobial therapy for ICU patients.

## Figures and Tables

**Table 1:** Antimicrobial susceptibility of Enterobacterales isolates from ICU and non-ICU patients from US hospitals (2018–2020).

Organism/	% Susceptible (No. of Isolates Tested) ^a^		
Antimicrobial Agent	ICU	VAP	Non-ICU
Enterobacterales	(4117)	(630)	(17,879)
Aztreonam/avibactam	100.0/>99.9 ^b^	100.0/99.8 ^b^	>99.9/99.9 ^b^
Ceftazidime/avibactam	99.7	99.5	99.9
Ceftolozane/tazobactam	90.1	86.6	95.5
Meropenem/vaborbactam	99.7	99.8	99.9
Imipenem/relebactam	94.4 ^c^	96.7 ^c^	91.9 ^c^
Piperacillin/tazobactam	82.4	78.5	90.5
Ceftazidime	81.7	81.2	88.1
Ceftriaxone	77.2	76.2	84.6
Meropenem	98.0	97.9	99.3
Imipenem	92.1	94.1	89.8
Levofloxacin	84.8	89.3	83.5
Gentamicin	91.3	93.3	92.1
Amikacin	94.9	96.5	95.1
Tigecycline	96.6	95.5	95.5
Colistin	77.0 ^d^	77.1 ^d^	78.6 ^d^
CRE	(78)	(15)	(117)
Aztreonam/avibactam	100.0/98.7 ^b^	100.0/100.0 ^b^	98.3/96.6 ^b^
Ceftazidime/avibactam	88.5	93.3	82.9
Meropenem/vaborbactam	82.1	93.3	81.2
Imipenem/relebactam	78.2 ^c^	93.3 ^c^	72.6 ^c^
Cefiderocol	92.3	100.0	94.9
Levofloxacin	38.5	53.3	32.5
Gentamicin	60.3	60.0	64.1
Amikacin	69.2	73.3	65.8
Tigecycline	96.2	86.7	94.9
Colistin	83.3 ^d^	73.3 ^d^	82.1 ^d^
MDR Enterobacterales	(979)	(149)	(3057)
Aztreonam/avibactam	100.0/99.8 ^b^	100.0/100.0 ^b^	99.9/99.7 ^b^
Ceftazidime/avibactam	98.7	98.0	99.1
Ceftolozane/tazobactam	58.6	43.6	73.7
Meropenem/vaborbactam	98.6	99.3	99.3
Imipenem/relebactam	96.1 ^c^	97.2 ^c^	96.4 ^c^
Piperacillin/tazobactam	36.8	22.1	52.6
Ceftazidime	26.8	23.5	37.3
Ceftriaxone	15.4	8.7	27.5
Meropenem	91.7	91.3	96.0
Imipenem	90.0	89.3	93.2
Levofloxacin	56.6	68.5	45.0
Gentamicin	68.9	77.2	63.4
Amikacin	86.1	93.3	85.1
Tigecycline	96.3	92.6	96.4
Colistin	87.3 ^d^	88.6 ^d^	90.8 ^d^
*P. aeruginosa*	(1304)	(308)	(2770)
Aztreonam/avibactam	78.0 ^e^	75.6 ^e^	81.9 ^e^
Ceftazidime/avibactam	96.3	95.8	97.6
Ceftolozane/tazobactam	97.2	97.1	98.4
Meropenem/vaborbactam	90.0 ^f^	87.0 ^f^	94.3 ^f^
Imipenem/relebactam	97.1	95.5	98.0
Piperacillin/tazobactam	77.8	75.6	84.6
Ceftazidime	81.4	80.8	87.8
Cefepime	84.7	83.1	89.0
Meropenem	76.9	70.8	85.8
Imipenem	77.4	73.7	84.3
Levofloxacin	73.3	68.3	73.2
Tobramycin	92.1	90.9	92.1
Colistin	99.8 ^d^	100.0 ^d^	99.7 ^d^

^a^ Susceptibility per CLSI and/or US FDA criteria [14,15]. ^b^ % Inhibited at ≤8/≤4 mg/L (PK/PD and EUCAST susceptible breakpoints, respectively). ^c^ All Enterobacterales species were included in the analysis, but CLSI excludes *Morganella*, *Proteus*, and *Providencia* species. ^d^ % Intermediate or not resistant. ^e^ % inhibited at ≤8 mg/L. ^f^ This compound is not approved for treatment of *P. aeruginosa* infections in the US. EUCAST criteria were applied since neither CLSI nor US FDA have published breakpoint criteria.

**Table 2:** Antimicrobial susceptibility of most common Enterobacterales species isolated from ICU and non-ICU patients from US hospitals (2018–2020).

Organism/	% Susceptible (No. of Isolates Tested) ^a^		
Antimicrobial Agent	ICU	VAP	Non-ICU
*K. pneumoniae*	(949)	(145)	(3581)
Aztreonam/avibactam	100.0/100.0 ^b^	100.0/100.0 ^b^	100.0/100.0 ^b^
Ceftazidime/avibactam	99.7	100.0	99.7
Ceftolozane/tazobactam	92.8	93.1	96.0
Meropenem/vaborbactam	99.4	100.0	99.7
Imipenem/relebactam	98.8 ^c^	100.0 ^c^	99.1 ^c^
Piperacillin/tazobactam	79.6	80.0	87.8
Ceftazidime	80.2	84.8	86.1
Ceftriaxone	79.8	82.8	85.6
Meropenem	96.4	97.9	98.2
Imipenem	97.0	98.6	98.2
Levofloxacin	83.3	85.5	86.7
Gentamicin	89.9	92.4	92.2
Amikacin	96.1	97.2	98.3
Tigecycline	97.7	93.1	98.2
Colistin	97.5 ^d^	97.9 ^d^	98.0 ^d^
*E. coli*	(898)	(103)	(6367)
Aztreonam/avibactam	100.0/100.0 ^b^	100.0/100.0 ^b^	>99.9/99.9 ^b^
Ceftazidime/avibactam	100.0	100.0	99.9
Ceftolozane/tazobactam	97.5	99.0	98.9
Meropenem/vaborbactam	100.0	100.0	99.9
Imipenem/relebactam	100.0 ^c^	100.0 ^c^	99.9 ^c^
Piperacillin/tazobactam	92.3	89.2	95.3
Ceftazidime	82.6	85.4	89.3
Ceftriaxone	79.1	82.5	87.0
Meropenem	99.9	100.0	99.9
Imipenem	100.0	100.0	99.8
Levofloxacin	71.7	79.6	76.1
Gentamicin	86.0	88.3	89.9
Amikacin	90.3	94.2	91.8
Tigecycline	100.0	100.0	100.0
Colistin	99.3 ^d^	100.0 ^d^	99.7 ^d^
*E. cloacae*	(515)	(96)	(1498)
Aztreonam/avibactam	100.0/99.8 ^b^	100.0/100.0 ^b^	100.0/99.9 ^b^
Ceftazidime/avibactam	98.6	99.0	99.7
Ceftolozane/tazobactam	69.3	57.3	80.5
Meropenem/vaborbactam	98.6	100.0	99.9
Imipenem/relebactam	97.7 ^c^	98.5 ^c^	99.9 ^c^
Piperacillin/tazobactam	63.8	54.2	74.9
Ceftazidime	62.7	53.1	73.6
Ceftriaxone	59.8	50.0	69.6
Meropenem	97.1	96.9	98.8
Imipenem	96.5	95.8	97.9
Levofloxacin	93.2	97.9	93.2
Gentamicin	95.7	99.0	96.7
Amikacin	98.4	99.0	99.1
Tigecycline	97.9	96.9	98.1
Colistin	79.7 ^d^	80.2 ^d^	80.4 ^d^

^a^ Susceptibility per CLSI and/or US FDA criteria [14,15]. ^b^ % Inhibited at ≤8/≤4 mg/L (PK/PD and EUCAST susceptible breakpoints, respectively). ^c^ All Enterobacterales species were included in the analysis, but CLSI excludes *Morganella*, *Proteus*, and *Providencia* species. ^d^ % Intermediate or not resistant.

**Table 3:** Cross-resistance among β-lactamases inhibitor combinations when testing Enterobacterales.

	Susceptibility by Resistance Phenotype (No. of Isolates)		
Antimicrobial Agent	Ceftazidime/Avibactam-Resistant (39) ^a^	Meropenem/Vaborbactam Non-Susceptible (36) ^a^	Imipenem/Relebactam-Resistant (225) ^a^
Aztreonam/avibactam	97.4/87.2 ^b^	100.0/94.4 ^b^	100.0/99.6 ^b^
Ceftazidime/avibactam	0.0	33.3	88.9
Meropenem/vaborbactam	38.5	0.0	85.3
Imipenem/relebactam	20.0	8.3	0.0

^a^ Including ICU and non-ICU isolates. ^b^ % Inhibited at ≤8/≤4 mg/L (PK/PD and EUCAST susceptible breakpoints, respectively).

## Data Availability

The datasets presented in this article are not readily available because the data are part of ongoing investigations. Requests to access the datasets should be directed to the corresponding author.
